# Surface Electroactive Sites of Tungstated Zirconia Catalysts for Vanadium Redox Flow Batteries

**DOI:** 10.1021/acsami.3c14633

**Published:** 2024-02-05

**Authors:** Aknachew Mebreku Demeku, Daniel Manaye Kabtamu, Guan-Cheng Chen, Yun-Ting Ou, Zih-Jhong Huang, Tai-Chin Chiang, Hsin-Chih Huang, Chen-Hao Wang

**Affiliations:** † Department of Materials Science and Engineering, National Taiwan University of Science and Technology, Taipei 106335, Taiwan; ‡ Department of Chemistry, Debre Berhan University, Po.Box: 445, Debre Berhan 00000, Ethiopia; § Global Development Engineering Program, National Taiwan University of Science and Technology, Taipei 106335, Taiwan; ∥ Hierarchical Green-Energy Materials (Hi-GEM) Research Center, National Cheng Kung University, Tainan 70101, Taiwan; ⊥ Advanced Manufacturing Research Center, National Taiwan University of Science and Technology, Taipei 106335, Taiwan

**Keywords:** metal−organic frameworks, vanadium redox flow batteries, tungstated zirconia, electrocatalysts, double-solvent method

## Abstract

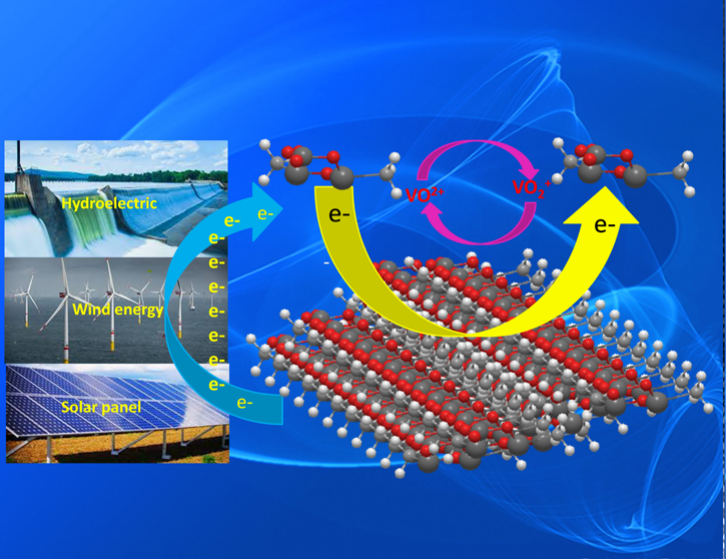

Surface electroactive sites for tungstate zirconia (WZ) were created by utilizing tungstate-immobilized UiO-66 as precursors via a double-solvent impregnation method under a mild calcination temperature. The WZ-22-650 catalyst, containing a moderate W content (22%), demonstrated a high density of surface electroactive sites. Proper heat treatment facilitated the binding of oligomeric tungsten clusters to stabilized tetragonal ZrO_2_, resulting in improved catalytic performance toward the VO^2+^/VO_2_^+^ redox couples compared to other tested samples. The substantial surface area, mesoporous structure, and establishment of new W–O–Zr bonds affirm the firm anchoring of WO_*x*_ to ZrO_2_. This robust attachment enhances surface electroactive sites, elevating the electrochemical performance of vanadium redox flow batteries (VRFBs). Charge–discharge tests further demonstrate that the superior voltage efficiency (VE) and energy efficiency (EE) for VRFBs using the WZ-22-650 catalyst are 87.76 and 83.94% at 80 mA cm^–2^, which are 13.42% VE and 10.88% EE better than heat-treated graphite felt, respectively. Even at a higher current density of 160 mA cm^–2^, VRFBs utilizing the WZ-22-650 catalyst maintained considerable efficiency, recording VE and EE values of 76.76 and 74.86%, respectively. This facile synthesis method resulted in WZ catalysts displaying superior catalytic activity and excellent cyclability, offering a promising avenue for the development of metal-oxide-based catalysts.

## 1 Introduction

Due to the continuing uncontrolled usage of fossil fuels, there is currently an increase in the energy crisis and environmental issues.^1^ With their cleanliness and renewability, renewable energy sources like tidal energy, wind, and solar have been hailed as the best replacement for fossil fuels.^2^ However, intermittent renewable energy is a significant barrier to effective integration into electric networks. To tackle this issue, the implementation of an energy storage system has been utilized.^1,3^ Among the various storage technologies that are currently accessible, redox flow batteries (RFBs) distinguish themselves due to their extended cycle life, fast response, pliable design, decoupled power and energy, and good safety features^1,4^ Among different RFBs, vanadium redox flow batteries (VRFBs) possess drawn widespread interest regarding their prospects for commercialization.^5,6^ VRFBs employ the redox couples of VO_2_^+^/VO^2+^ and V^3+^/V^2+^ on the positive and negative sides of electrolytes with sulfuric acid as medium, respectively, which successfully prevent cross-contamination of metal ions through the membrane.^1,3,7^

The electrode, which makes up the majority of VRFBs, directly affects the battery performance since it offers places for redox processes to occur and pathways for mass transfer and electron conduction.^7,8^ Due to its enormous porosity, good electrical conductivity, robust stability, good corrosion resistance, and low cost, graphite felt (GF) is a popular choice for use as carbon-based electrode material in VRFBs.^7,9^ However, the limited specific surface area mainly causes the low energy efficiency and poor electrochemical activity of VRFBs, poor hydrophilicity, insufficient active sites, and poor chemical kinetics of graphite felt (GF).^5,7,10^ To improve the performance of GF electrodes toward VRFBs, various treatments such as acid,^11^ thermal, electrochemical oxidation,^12^ ammoxidation reaction, plasma, and modification with metals or metal oxides have been proposed.^1,5,7,10−13^ These modifications improve the electrochemical performance by increasing the number of active sites and hydrophilicity. Metal-based (metal oxides, metal carbides, and metal nitrides) catalysts greatly enhance the electrochemical performance of vanadium redox reactions. Precious metals such as Pt, Au, Ir, Cu, and Bi exhibit elevated electrochemical reactivity and exceptional conductivity, and possess effective resistance against corrosive acidic electrolytes.^10,14^ Nevertheless, precious metals have lower availability, easy access to side reactions, low mechanical stability, and higher costs; therefore, they are not suitable for the potential application of VRFBs.^15^ Different electrochemically active metal oxides (ZrO_2_, WO_3_, SnO_2_, Nb_2_O_5_, CeO_2_, Ta_2_O_5_, TiO_2_, W_18_O_49_,^16^ Mn_3_O_4_, PbO_2_, etc.) have garnered increasing interest because of their superior catalytic activity and inexpensive cost to improve VRFBs performances.^1,10,16−19^ However, the progress of metal oxides is impeded by their insufficient electrical conductance, feeble amalgamation, inferior dispersal, and challenging nanocrystallization.^1,20^

To address this problem, metal–organic frameworks (MOFs) have drawn considerable focus in catalysts because of their adjustable structure, high porosity,^21^ high thermal and chemical stability,^22^ and ease of functionalization.^21−23^ MOFs have a stable structure comprising metal-based nodes and a coordination network with organic linkers, including potential voids.^4,24^ These characteristics, exceptional porosity, and lack of concealed spaces within the frameworks inherently make them valuable for practical uses such as separations, purification, adsorption, and catalytic applications. Furthermore, MOFs feature a structured alignment of metal nodes and heteroatoms, making them prone to the creation of evenly dispersed metal species and additional dopants.^25^

Catalytic activity arising from molecular moieties and various MOFs demonstrates better catalytic activity sources from metal ions. By eliminating solvent ligands, inorganic nodes have the potential to exhibit catalytic activity, leading to the formation of harmoniously unsaturated metal ion sites that serve as centers for catalysis.^26^ These molecules with catalytic activity can be postgrafted onto the framework after forming MOFs or during the synthesis process, integrated directly into MOFs by being preattached onto the organic linkers. Inspired by this, UiO-66, a type of zirconium-containing MOFs, serves as a precursor for ZrO_2_ and morphological templates, designed for the synthesis of ZrO_2_ through thermal decomposition in ambient air.^27^ Moreover, the substantial porosity and hydrophilic pore surface of UiO-66 permit the utilization of the double-solvent technique for the entrapment of hydrophilic guest species (such as ammonium meta-tungstate) within its pores as well.^28^

We proposed UiO-66 as a practical precursor for the synthesis of tungsten oxide/zirconium dioxide (WO_*x*_/ZrO_2_, tungstated zirconia, denoted as WZ) and employed it as an electrocatalyst material for VRFBs. We have successfully prepared WZ catalysts through the double-solvent impregnation method followed by the pyrolysis of tungstate-immobilized UiO-66 in the air. Because of the larger surface area and carbon porosity, crucial for boosting vanadium redox reactions, the synthesized MOF-derived WZ-decorated GF electrode demonstrates superior electrochemical performance toward VRFBs. A single cell using WZ-22-650-modified heat-treated graphite felt (HGF) yielded a high voltage efficiency (VE) and energy efficiency (EE) of 87.76 and 83.94%, respectively, at a current density of 80 mA cm^–2^, which is 13.42% VE and 10.88% EE more efficient than heat-treated graphite felt.

## 2 Experimental Part

### 2.1 UiO-66 Synthesis

In a typical procedure, 1.40 g of ZrOCl_2_·8H_2_O and 1.02 g of BDC were mixed in 62 mL of DMF. The mixture was moved to a hydrothermal reactor and heated at 120 °C for 24 h. After cooling to ambient temperature, activation occurs via cascade reflux with DMF and methanol. The resulting UiO-66 metal–organic framework (MOF) powders are vacuum-sealed at 130 °C.^28^

### 2.2 Synthesis of WO_3_

3 mmol of Na_2_WO_4_·2H_2_O was dissolved in 20 mL of deionized water and mixed with 10 mL of dilute HCl. After stirring for 40 min, the solution was moved to a hydrothermal reactor and heated at 200 °C for 24 h. The resulting WO_3_ sample was filtered, rinsed with distilled water and ethanol, and then dried at 60 °C for 10 h.

### 2.3 WO_*x*_/UiO-66 Synthesis

WO_*x*_/UiO-66 was prepared via double-solvent impregnation. Typically, 800 mg of UiO-66 was dissolved in 60 mL of dry *n*-hexane and sonicated for 15 min until a uniform solution was obtained. After 2 h of stirring at 50 °C, 0.8 mL of varied concentrations of aqueous ammonium meta-tungstate solution was added dropwise over a 15 min interval of steady, vigorous swirling.^28^ For 8 h, the resultant solution was stirred continually and dried at 100 °C after meticulous filtration. To optimize WO_*x*_/UiO-66, we added 10, 22, and 65 mg of ammonium meta-tungstate. The obtained WO_*x*_/UiO-66 were calcined in the air for 6 h at 650 °C, and the samples were denoted as tungstate zirconia (WZ) WZ-10-650, WZ-22-650, and WZ-65-650, respectively. A similar process prepared UiO-66 without ammonium meta-tungstate and ZrO_2_, denoted as WZ-0-650, for comparison. Scheme S1 illustrates the general synthetic procedure of WO_*x*_/UiO-66 into tungstate zirconia (WZ).

Field emission scanning electron microscopy (JSM-6500F) examined morphology. Transmission electron microscopy (FEI Tecnai G2 F-20 S-TWIN) coupled with elemental energy-dispersive spectroscopy (EDS) mapped microstructures. X-ray diffraction (Bruker D2) with a Cu Kα radiation source of λ = 1.54 Å determined phase and crystalline structure. Raman spectrometer (iHR550) with a 532 nm laser assessed molecular vibration states and structural faults. X-ray photoelectron spectroscopy (Thermo, K-Alpha) analyzed the surface composition and bonding. Brunauer–Emmett–Teller (NOVA touch LX^2^) analysis measured specific surface area and porosity. Contact angle measurement (FTA-125) evaluated the material wettability.

Cyclic voltammetry (CV) was measured using a standard three-electrode setup and an electrochemical workstation (Bio-Logic, VSP-300) in a cell with three electrodes at ambient temperature. A glassy carbon ring disk electrode (RDE) served as the working electrode, while platinum wire and Ag/AgCl were utilized as the counter and reference electrodes, respectively. The RDE ink consisted of 7.5 mg of catalyst, 2.8 mL of isopropanol, 2.8 mL of deionized water, and 0.04 mL of 5% Nafion solution. The electrolyte was 1.6 M VOSO_4_ + 4.6 M H_2_SO_4_, and N_2_ purging minimized unwanted species oxidation. The potential range was 0–1.5 V, with a scan rate of 10 mV s^–1^. Electrochemical impedance spectroscopy (EIS) was performed in the 100 kHz to 10 mHz frequency range at 1.0 V. For the graphite felt (Ce Tech Co., Ltd.) electrode, CV testing involved a circular GF (geometric area of 1.327 cm^2^) as the working electrode, connected via a gold wire to the equipment. The electrolyte used was 0.05 M VOSO_4_ in 2 M H_2_SO_4_. CV parameters were 0.0–1.5 V and a scan rate of 5 mV s^–1^, with EIS using an AC voltage of 10 mV. Nitrogen gas purging was employed during CV experiments to protect undesired reactions.

VRFBs single-cell experiments were conducted in a solution containing 1.6 M VOSO_4_ and 4.6 M H_2_SO_4_. A graphite felt (5.00 cm × 5.00 cm × 0.65 cm) embedded with a MOF-derived catalyst served as the positive electrode, while heat-treated graphite felt (HGF) was employed as the negative electrode. Nafion 212 ion exchange membranes were situated between the cell frames. Each electrolyte storage chamber had a volume of 60 mL and was individually circulated at 80 mL min^–1^ using FMI pumps and nitrogen gas was purged on the negative side. Charge/discharge potential ranged between 0.7 and 1.6 V, and different current densities (80, 100, 120, 140, and 160 mA cm^–2^) were used for measurements. The detailed formulation of Coulombic efficiency (CE), voltage efficiency (VE), and energy efficiency (EE) were outlined in the previous report.^20,29^

The process of fabricating a WZ catalyst electrode was on a graphite felt substrate. To fabricate the HGF-WZ-22-650 electrode, 25 mg of the sample was combined with a mixture of 40 mL of ethanol and 5 mL of 5 wt % Nafion. The resulting mixture was ultrasonicated for 1 h to achieve a fully dispersed suspension. Afterward, the heat-treated graphite felt was immersed in ink and subjected to ultrasonication for 5 min. Afterward, the treated graphite felt was put in an oven set at a temperature of 80 °C for 30 min. This step was continued until the entire electrolyte was fully utilized. Once the ink-drying process was completed, the graphite felt was allowed to dry at 80 °C for 24 h in a vacuum oven. The general experimental process of incorporation of WZ catalyst on graphite felt is illustrated in Scheme S2.

## 3 Results and Discussion

**Figure 1:**
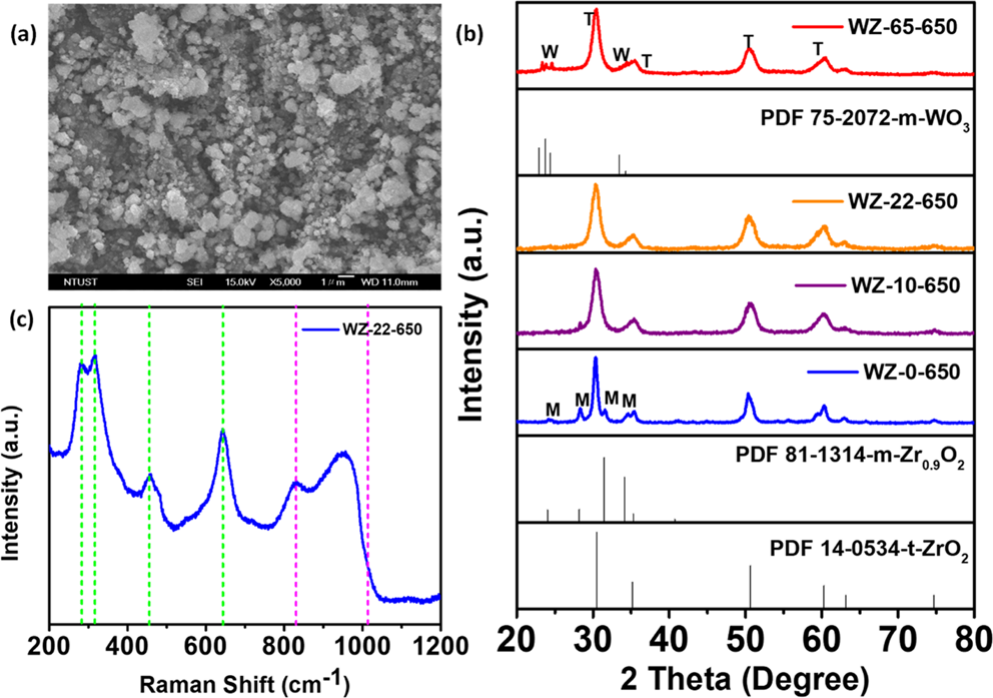
(a) FE-SEM image, (b) XRD patterns of different W contents: (t-ZrO_2_: tetragonal, m-ZrO_2_: monoclinic, and monoclinic tungsten phase: m-WO_3_), and (c) WZ Raman spectrum of WZ at 650 °C.

FESEM images of the prepared WZ demonstrate that the WZ-22-650 °C catalyst comprises grape cluster-shaped particles (Figures 1a and S1b). The UiO-66 zirconium MOF image is depicted in Figure S1a. The results of the WZ XRD pattern with different W contents are shown in Figures 1b and S2d. The UiO-66, WO_3_, and WZ-0-650 patterns are shown in Figures S2a–c, respectively. The WZ-0-650 exhibits both tetragonal and monoclinic structures. Compared to pure zirconia oxide, WZ has very distinct crystalline structures, and adding W significantly improves the fraction of t-ZrO_2_ in hybrid materials. Mixed phases of t-ZrO_2_ and m-ZrO_2_ are seen for the sample WZ-10-650 (10% W in weight), but t-ZrO_2_ predominates. The WZ-22-650 (22% W in weight) and WZ-65-650 (65% W in weight) samples show only one phase of t-ZrO_2_. This occurrence demonstrates the stabilizing impact of the WO_*x*_ species on t-ZrO_2_ at the specified temperature (650 °C) by suppressing the transformation of t-ZrO_2_ to m-ZrO_2_.^28,30^ The outcome of the double-solvent approach suggests that highly diffused surface WO_*x*_ species have a stabilizing effect on the structure of ZrO_2_. The three diffraction XRD patterns 2θ between 23 and 25° correspond to the monoclinic microcrystallites of WO_3_ that are formed by the aggregation of WO_*x*_ species on the surface of zirconia. These patterns vividly illustrate the growth of crystalline WO_3_ as the concentration of W increases to 65% (WZ-65-650).^28^

The Raman spectrum of a WZ catalyst with a W concentration of 22% at 650 °C is presented in Figure 1c in the range 200–1200 cm^–1^. The spectrum exhibits distinctive peaks corresponding to t-ZrO_2_, with notable bands detected at 267, 315, 458, and 643 cm^–1^.^28,31^ Furthermore, the spectral graph pertaining to the supported WO_*x*_ species can be detected in the range of 800–1100 cm^–1^. However, the earlier report does not support the Raman characteristics of ZrO_2_.^28,32^ A band of vibration at 1000 cm^–1^ is caused by the stretching vibration of the W=O double bonds, and mono- and poly-tungsten species bonds exist. When the frequency increases, new shoulder peaks develop, attributed to geometrically distinct WO_*x*_ species on ZrO_2_. The W=O vibration mode in the supported WO_*x*_ species is found in the band around 970 cm^–1^. The Raman shift at approximately 910 cm^–1^ is provisionally correlated with W–O–Zr stretching vibrations, suggesting the immobilized WO_*x*_ species on ZrO_2_. Simultaneously, the band around 830 cm^–1^, typically indicative of W–O–W stretching vibrations, indicates the likelihood of the surface WO_*x*_ species existing as compact oligomeric clusters, rather than larger structures.^28,33^

**Figure 2:**
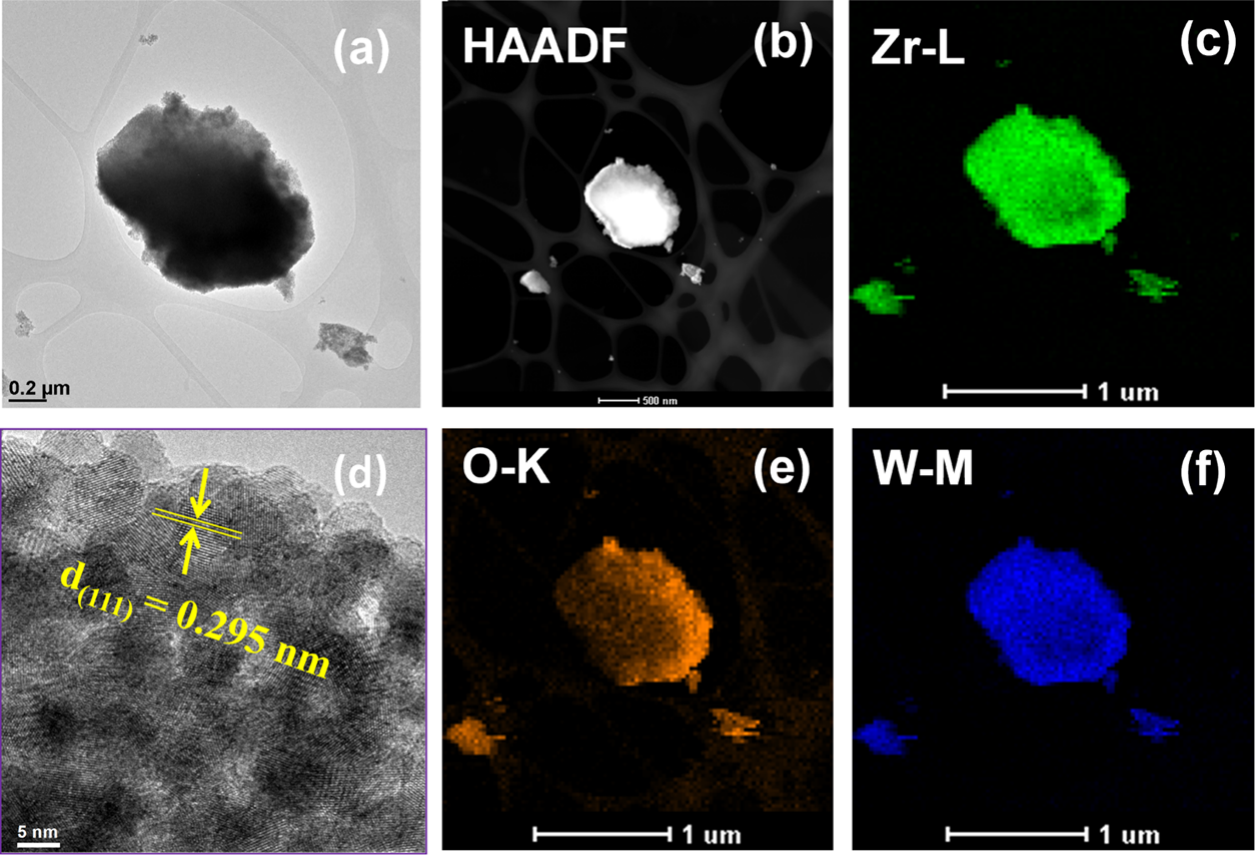
(a) TEM, (b) HAADF-STEM, (c, e, f) chemical mapping, and (d) HR-TEM images for the calcined WZ-22-650 catalyst.

The TEM image of the WZ-22-650 catalyst demonstrates the creation of particles that possess a rectangular platelike morphology (Figure 2a) and the HAADF-STEM image (Figure 2b). The image obtained from high-resolution transmission electron microscopy (HR-TEM) reveals the presence of the t-ZrO_2_ phase calcined at 650 °C, as depicted in Figure 2d. The interplanar spacing is estimated to be 0.295 nm. These values are aligned with the XRD results and correspond to the *d*-spacing of the (111) plane of t-ZrO_2_.^34^ The chemical elemental mapping by EDS analysis indicates the coexistence of W, O, and Zr, as shown in Figure 2c,e,f also reveals their uniform distribution throughout the sample.^34,35^

**Figure 3:**
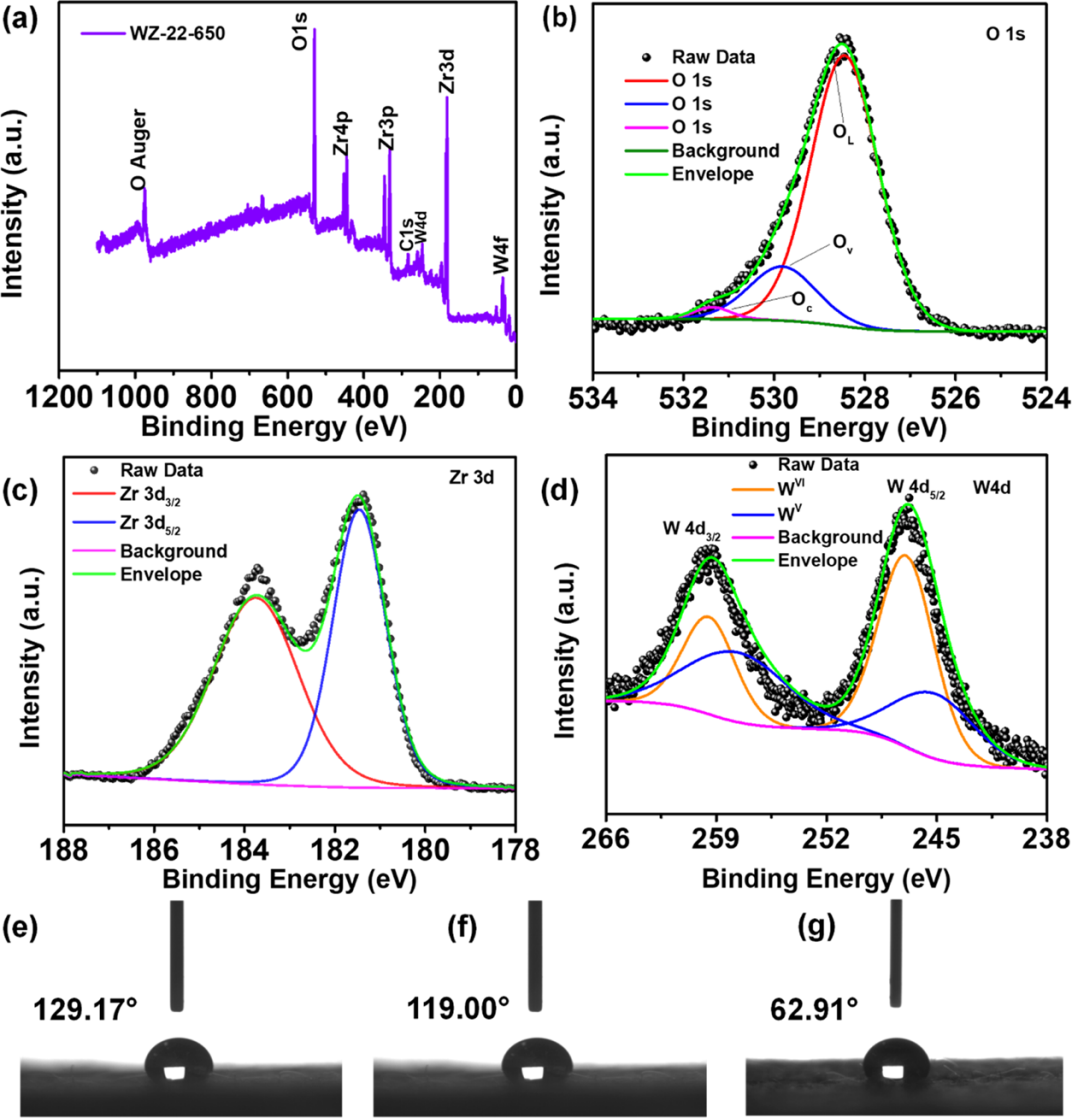
XPS analysis of the WZ-22-650: (a) element survey, (b) O 1s, (c) Zr 3d, and (d) W 4f. Wettability measurements in (e) PGF, (f) HGF, and (g) HGF-WZ-22-650.

XPS was used to detect the surface chemical states and composition of elements of the WZ-22-650. The survey spectrum reveals the presence of W, O, C, and Zr in the WZ-22-650 (Figure 3a). The spectrum of O 1s is depicted in Figure 3b, consisting of a strong peak and two distinct shoulder peaks. Upon closer examination through fitting analysis, the O 1s spectra can be segmented into three peaks located at 528.5, 529.8, and 531.4 eV. These terms refer to the presence of oxygen in the lattice structure (O_L_), the vacancy of oxygen in the metal oxide material (O_v_), and the attachment of oxygen molecules on the surface (O_c_), respectively. In Figure 3c, the Zr 3d XPS spectrum displays two distinct peaks characterized by binding energies situated at 181.4 and 183.7 eV. These peaks correspond to Zr 3d_5/2_ and Zr 3d_3/2_, respectively, indicating the presence of Zr^4+^ oxidation states.^36^Figure 3d shows the W 4d spectrum, which has been effectively modeled using a doublet possessing binding energies measured at 247.01 and 259.5 eV. These energies can be associated with W 4d_5/2_ and W 4d_3/2_ levels, respectively, and they exhibit a spin–orbit splitting difference of 12.4 eV.^37^ Moreover, within the W 4d spectrum, two doublets are observed, corresponding to the oxidation states of W^VI^ and W^V^.^38^ Furthermore, the results of the XPS adjustment of WO_3_ and WZ-0-650 are depicted in Figure S3a–f.

The water droplet technique was employed to ascertain the contact angle of each sample and to examine the impact of surface modification on graphite felt (GF) hydrophilicity. The contact angles of water on pristine graphite felt (PGF), heat-treated graphite felt (HGF), and catalyst deposited on the heat-treated graphite felt (HGF-WZ-22-650) surfaces are 129.17, 119.00, and 62.91°, respectively (Figure 3e,g). Compared to PGF and HGF, the wettability of the HGF-WZ-22-650 electrode has increased significantly and has a much higher surface energy. The hydrophilicity of the surface is enhanced by the existence of functional groups containing oxygen, thereby creating a conducive environment for electrochemical processes.^19^ Moreover, the water contact angle test of WZ-0-650 and WO_3_, as shown in Figure S7.

**Figure 4:**
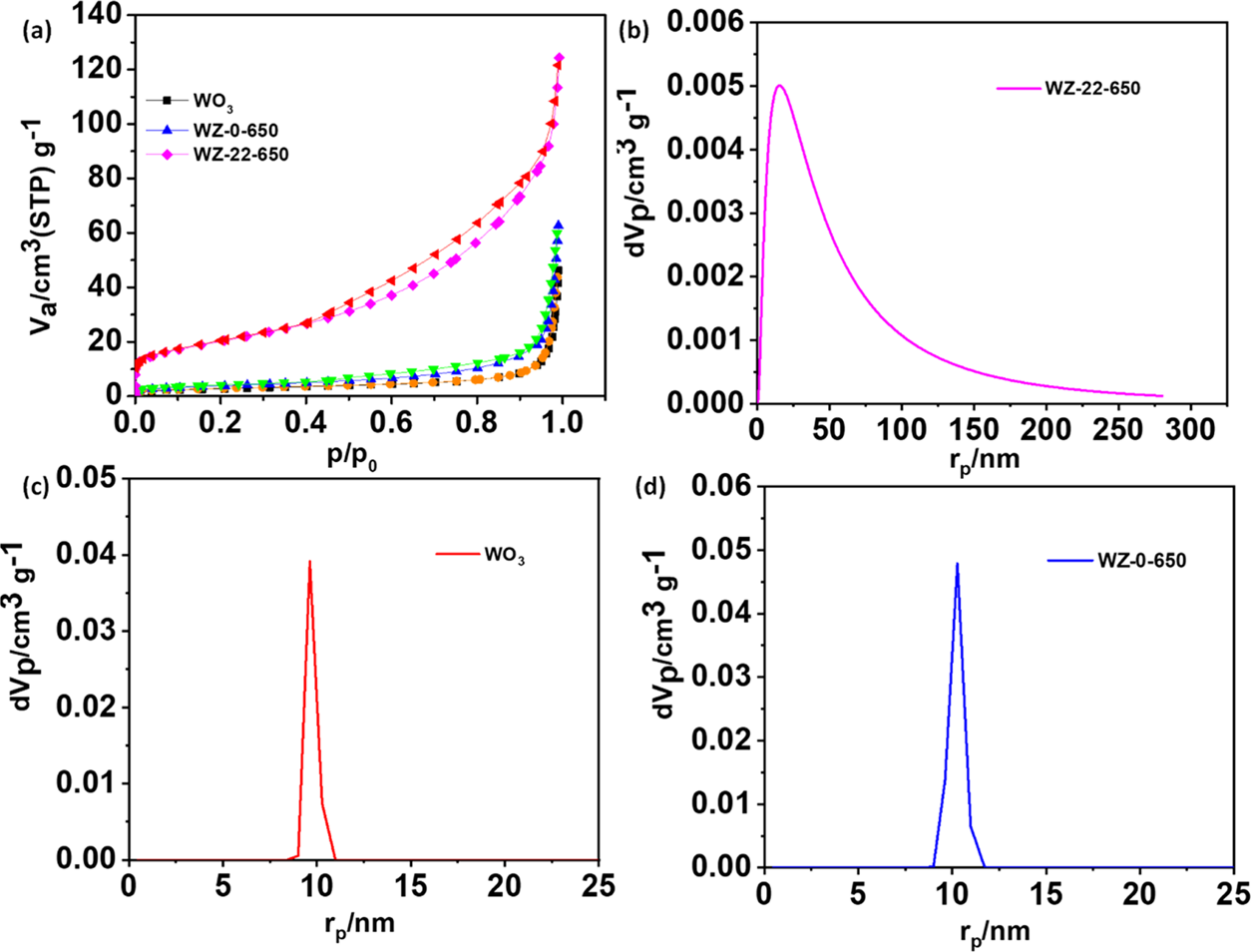
(a) N_2_ adsorption–desorption isotherm of WO_3_, WZ-0-650, and WZ-22-650. (b, c), (b–d) Pore diameters of the sample WZ-22-650, WO_3_, and WZ-0-650, respectively.

The N_2_ adsorption/desorption isotherms of the WO_3_, WZ-0-650, and WZ-22-650 samples obtained are shown in Figure 4a. The profiles of these samples display a type-IV isotherm (2–50 nm) and a substantial hysteresis loop, corresponding to the mesopores’ existence.^39^ The calculated surface areas are given in Table 1, as obtained from Figure 4b,d. The WZ-22-650 catalyst has more significant surface areas and total pore volumes than those of WO_3_ (Figure 4c) and WZ-0-650 (Figure 4d). A larger surface area gives greater surface electroactive sites, which improves the VRFB’s electrochemical performance. Moreover, the formation of new W–O–Zr bonds provides evidence that the WO_*x*_ species are firmly bonded to ZrO_2_ and that the surface areas of the resulting WZ material arise from both the inner and outer surfaces, as well as the interstitial spaces between the particles.^28,39^

**Table 1:** Lists of the Textural Characteristics of WZ-22-650, WZ-0-650, and WO_3_ Obtained from Figure 4

catalyst	surface area (m^2^ g^–1^)^a^	pore volume (cm^3^ g^–1^)^b^	mean pore diameter (nm)
WO_3_	9.76	0.0711	29.129
WZ-0-650	13.66	0.0959	28.095
WZ-22-650	122.86	0.1839	10.125

a Single-point pore volume determined at *P*/*P*_0_ = 0.89.

b Specific areas determined by the BET method.

**Figure 5:**
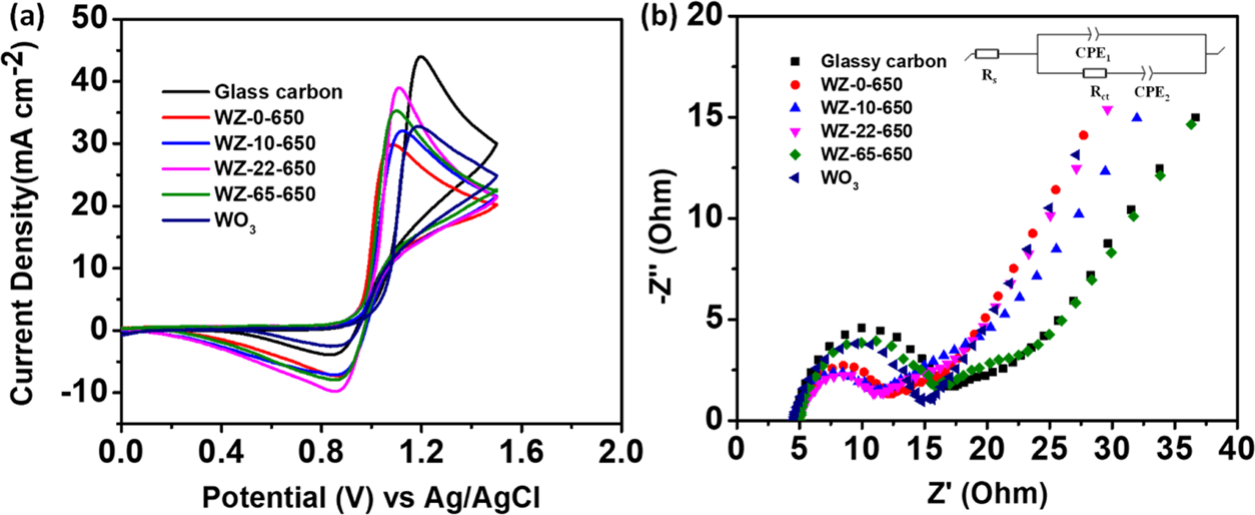
(a) CV curves and (b) Nyquist plots of GC, (WZ-0, WZ-10, WZ-22, and WZ-65)-650 in the electrolyte solutions were composed of 1.6 M VOSO_4_ + 4.6 M H_2_SO_4_ at a scan rate of 10 mV s^–1^.

The electrode’s electrochemical behavior was assessed, with Figure 5a displaying the cyclic voltammetry (CV) curves of the electrodes employing (WZ-0, WZ-10, WZ-22, and WZ-65)-650 and WO_3_. The detailed peak current density (*J*_pa_ and *J*_pc_) and peak potential separation (Δ*E*_p_) of electrochemical data obtained from Figure 5a are summarized in Table 2. The ordering of Δ*E*_p_ values for the samples in the VO^2+^/VO_2_^+^ redox reaction is as follows: WZ-0-650 < WZ-65-650 < WZ-22-650 < WZ-10-650 < WO_3_ < GC without a catalyst. Moreover, the redox peak current ratios (*I*_pa_/*I*_pc_) of the catalysts are arranged in ascending order, as follows: WZ-22-650 (1.81) < WZ-0-650 (1.99) < WZ-65-650 (2.07) < WZ-10-650 (2.2) < WO_3_ (2.45) < GC (4.26). However, WZ-22-650 displays the highest *J*_pa_ and *J*_pc_, as well as a lower redox peak current ratio compared with other electrodes. Accordingly, WZ-22-650 exhibits superior electrocatalytic activity for VO^2+^/VO_2_^+^ redox reactions. The superior catalytic efficacy exhibited by WZ-22–650 toward the vanadium redox couple can be ascribed to its extensive specific surface area and mesoporous configuration, which afford abundant active sites that augment the reaction.^40^ Furthermore, the creation of new W–O–Zr bonds confirms that WO_*x*_ is strongly anchored to ZrO_2_.^28,33^

**Table 2:** CV Test Results Obtained from Figure 5a

catalyst	*J*_pa_ (mA cm^–2^)	*J*_pc_ (mA cm^–2^)	*E*_pa_ (V)	*E*_pc_ (V)	Δ*E*_p_ (V)
GC	44.018	–3.526	1.195	0.840	0.355
WZ-0-650	29.840	–7.129	1.088	0.878	0.210
WO_3_	33.124	–2.301	1.182	0.884	0.298
WZ-10-650	32.083	–7.353	1.127	0.869	0.258
WZ-22-650	39.018	–9.772	1.1069	0.862	0.245
WZ-65-650	35.508	–7.924	1.095	0.859	0.236

Electrochemical impedance spectroscopy (EIS) was employed to assess the electrocatalytic activity of various electrodes during the VO^2+^/VO_2_^+^ redox process as shown in Figure 5b. All curves show a semicircle in the high-frequency zone and a linear one in the low-frequency region.^41^ This implies that the electrode reaction at the polarization potential is governed by the concurrent influence of the charge transfer and mass transfer processes. The resistance to charge transfer could be assessed using the diameter.^40,42^ The Nyquist plots displayed a high-frequency semicircle indicating charge transfer and a low-frequency linear portion suggesting vanadium ion diffusion.^41,43^ Furthermore, the Nyquist plots can be accurately represented by the inset equivalent circuit, where *R*_s_, *R*_ct_, CPE_1_, and CPE_2_ correspond to ohmic resistance, charge transfer resistance, diffusion capacitance, and electric double-layer capacitance, respectively. Table 3 and Figure 5b display the corresponding fitting electrochemical parameters (*R*_s_ and *R*_ct_) of glassy carbon (WZ-0, WZ-10, WZ-22, and WZ-65)-650, and WO_3_. While all electrodes showed similar *R*_s_ values (4.49–4.98 Ω), the WZ-22-650 electrode exhibited a smaller *R*_ct_, indicating superior electrochemical activity attributed to its larger specific surface area enhancing diffusion and charge transfer processes.^40^

**Table 3:** EIS Fitting Outcomes Data Derived from Figure 5b

catalyst	*R*_s_ (Ω)	*R*_ct_ (Ω)	CPE_1_ (F s^a–1^)	CPE_2_ (F s^a–1^)
glassy carbon	4.51	12.36	0.106 × 10^–3^ (*a* = 0.805)	0.1138 (*a* = 0.206)
WZ-0-650	4.86	7.99	0.294 × 10^–3^ (*a* = 0.726)	0.1363 (*a* = 0.423)
WO_3_	4.90	10.22	0.128 × 10^–3^ (*a* = 0.773)	0.1079 (*a* = 0.373)
WZ-10-650	4.49	6.01	0.190 × 10^–3^ (*a* = 0.773)	0.0829 (*a* = 0.327)
WZ-22-650	4.98	5.34	0.178 × 10^–3^ (*a* = 0.762)	0.1033 (*a* = 0.387)
WZ-65-650	4.80	8.30	0.416 × 10^–3^ (*a* = 0.678)	0.1077 (*a* = 0.389)

**Figure 6:**
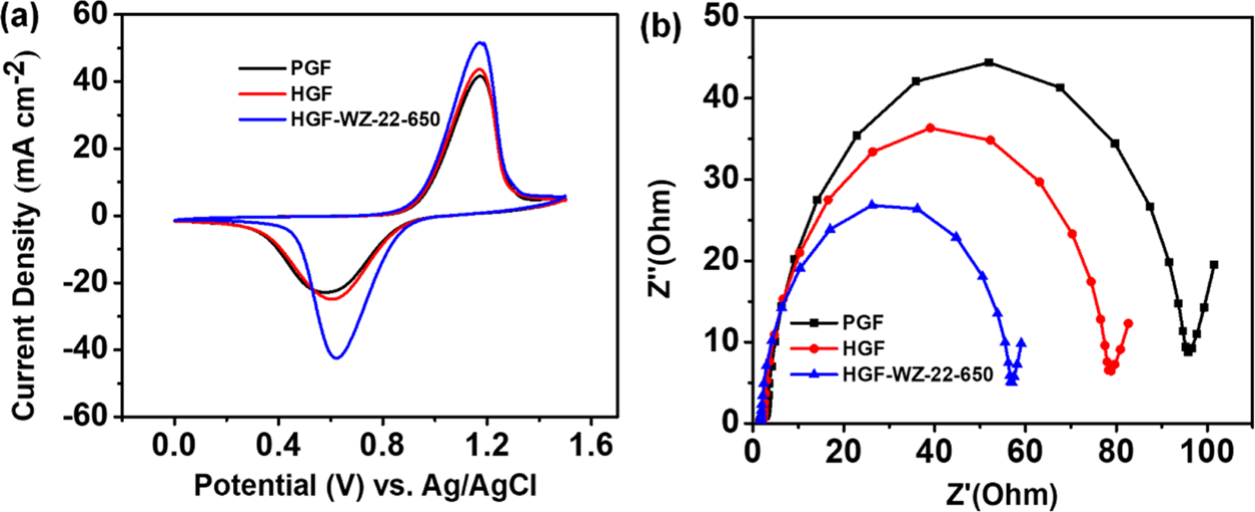
(a) CV curves and (b) Nyquist plots of PGF, HGF, and HGF-WZ-22-650.

The prepared PGF, HGF, and HGF-WZ-22-650 electrodes were subjected to CV and EIS tests to assess their electrochemical performance in the VO^2+^/VO_2_^+^ redox process (Figure 6). Figure 6a shows the CV plots for the three samples toward the VO^2+^/VO_2_^+^ redox pair, and Table 4 displays the data derived from the CV results. The HGF-WZ-22-650 electrode has the best electrochemical activity among the evaluated materials, exhibiting the smallest Δ*E*_p_ and the highest *J*_p_ with regard to the VO^2+^/VO_2_^+^ reaction. The enhanced catalytic activity of the HGF-WZ-22-650 electrode originates from its substantial concentration of active sites and large surface area, as demonstrated in Table 1. Figure 6b shows Nyquist plots of PGF, HGF, and HGF-WZ-22-650 in a 0.05 M VOSO_4_ + 2 M H_2_SO_4_ solution, using a 5 mV excitation signal across frequencies ranging from 100 kHz to 10 mHz in open-circuit potential (OCP). Each plot exhibits a high-frequency semicircle indicating charge transfer resistance and a low-frequency ray. Notably, the HGF-WZ-22-650 plot shows the smallest semicircle radius, indicating the lowest charge transfer resistance among the samples. Table 5 summarizes the specific values obtained from the fitting process.

**Table 4:** CV Test Results Obtained from Figure 6a

catalyst	*J*_pa_ (mA cm^–2^)	*J*_pc_ (mA cm^–2^)	*E*_pa_ (V)	*E*_pc_ (V)	Δ*E*_p_ (V)
PGF	41.808	–21.944	1.179	0.592	0.587
HGF	43.578	–24.599	1.171	0.611	0.560
HGF-WZ-22-650	51.773	–41.939	1.175	0.623	0.552

**Table 5:** EIS Results Obtained from Figure 6b

catalyst	*R*_s_ (Ω)	*R*_c_ (Ω)	CPE_1_ (FS^a–1^)	CPE_2_ (FS^a–1^)
PGF	1.65	103.1	2.402 × 10^–3^ (*a* = 0.881)	1.002 (*a* = 0.500)
HGF	1.69	80.88	2.698 × 10^–3^ (*a* = 0.878)	1.228 (*a* = 0.61)
HGF-WZ-22-650	1.47	57.48	3.517 × 10^–3^ (*a* = 0.925)	0.794 (*a* = 0.715)

**Figure 7:**
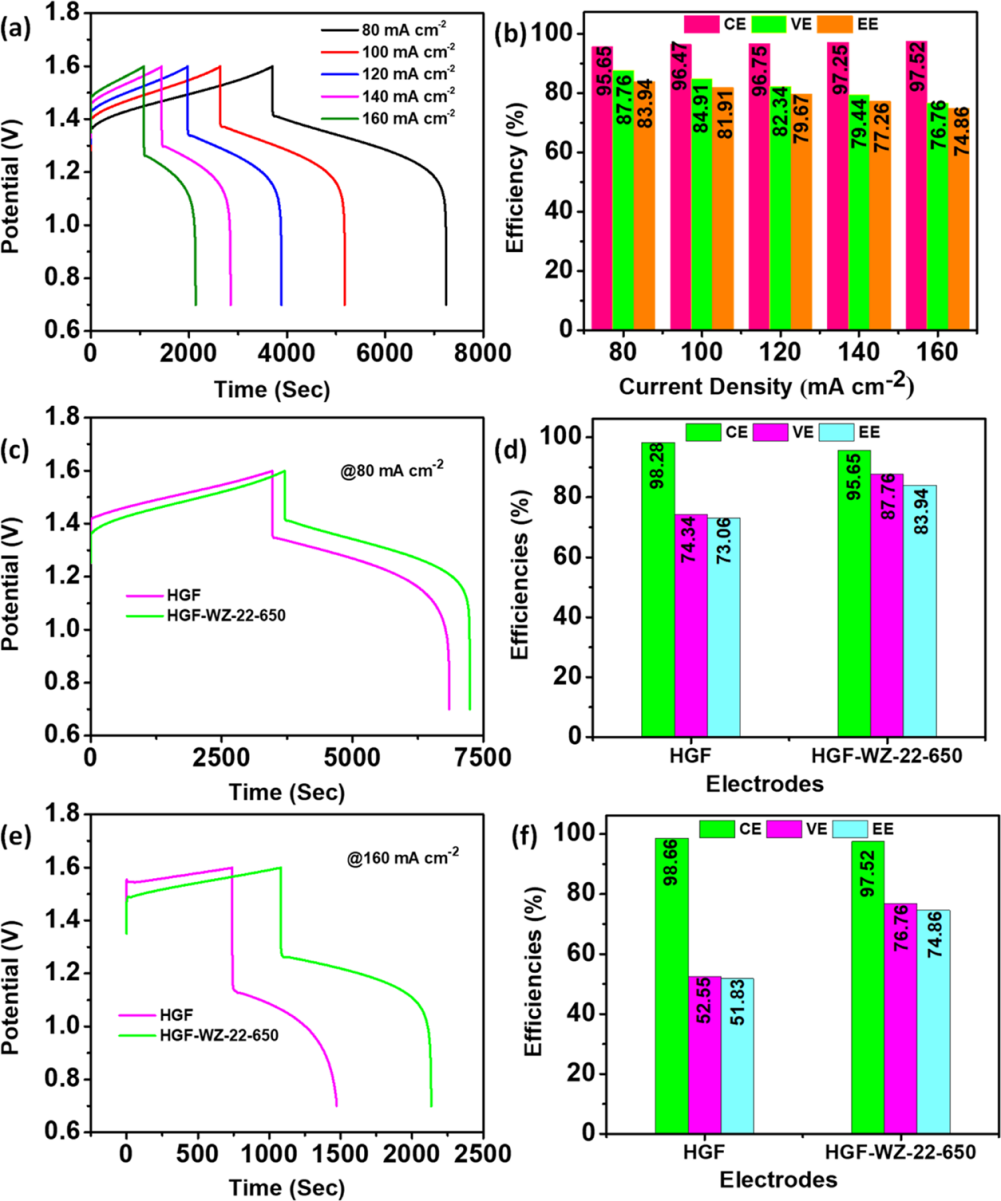
(a) HGF-WZ-22-650 charge/discharge curves at different *J*, (b) estimated efficiency values, (c, e) charge/discharge curves, and (d, f) efficiencies of the HGF and HGF-WZ-22-650 electrodes at 80 and 160 mA cm^–2^, respectively.

Figure 7a presents the charge/discharge patterns of the cell utilizing the HGF-WZ-22-650 electrode at a variety of current densities (*J*) ranging from 80 to 160 mA cm^–2^. The resulting efficiency outcomes are depicted in Figure 7b. The increase in *J* slightly augments the percentage of Coulombic efficiency (CE), ascribed to the shortened charge/discharge period, which results in a shorter time for metal ion crossover across a Nafion 212 membrane. Nevertheless, the expedited charging and discharging typically lead to an upsurge in ohmic resistance and overpotential, leading to a decrease in voltage efficiency (VE) and energy efficiency (EE).^3,18^ Cell charge/discharge voltage curves with HGF and HGF-WZ-22-650 electrodes were measured at the same *J* of 80 mA cm^–2^, as presented in Figure 7c. The HGF-WZ-22-650 electrode cell displays a lower reaction overpotential, extended discharge duration, reduced charge voltage, and higher discharge voltage compared to the HGF electrode during charge/discharge processes. Figure 7d shows the efficiency results obtained from Figure 7c. As shown in Figure 7d, the HGF-WZ-22-650 electrode achieves better CE, VE, and EE values of 95.65, 87.76, and 83.94%, respectively, at 80 mA cm^–2^, which is 13.42% VE and 10.88% EE more efficient than those of HGF. Furthermore, Figure 7f displays the efficiency results obtained from Figure 7e. As shown in Figure 7e, the HGF-WZ-22-650 electrode achieves better CE, VE, and EE values of 97.52, 76.76, and 74.86%, respectively, at a higher *J* of 160 mA cm^–2^. Enhanced cell performance stems from the even distribution of WZ-22-650 nanoparticles on the GF fiber surfaces, leading to increased oxygen-containing functional groups. These groups create additional active sites for adsorbing energy-containing redox couples (VO^2+^/VO_2_^+^), thereby improving both electrolyte accessibility and cell performance.

**Figure 8:**
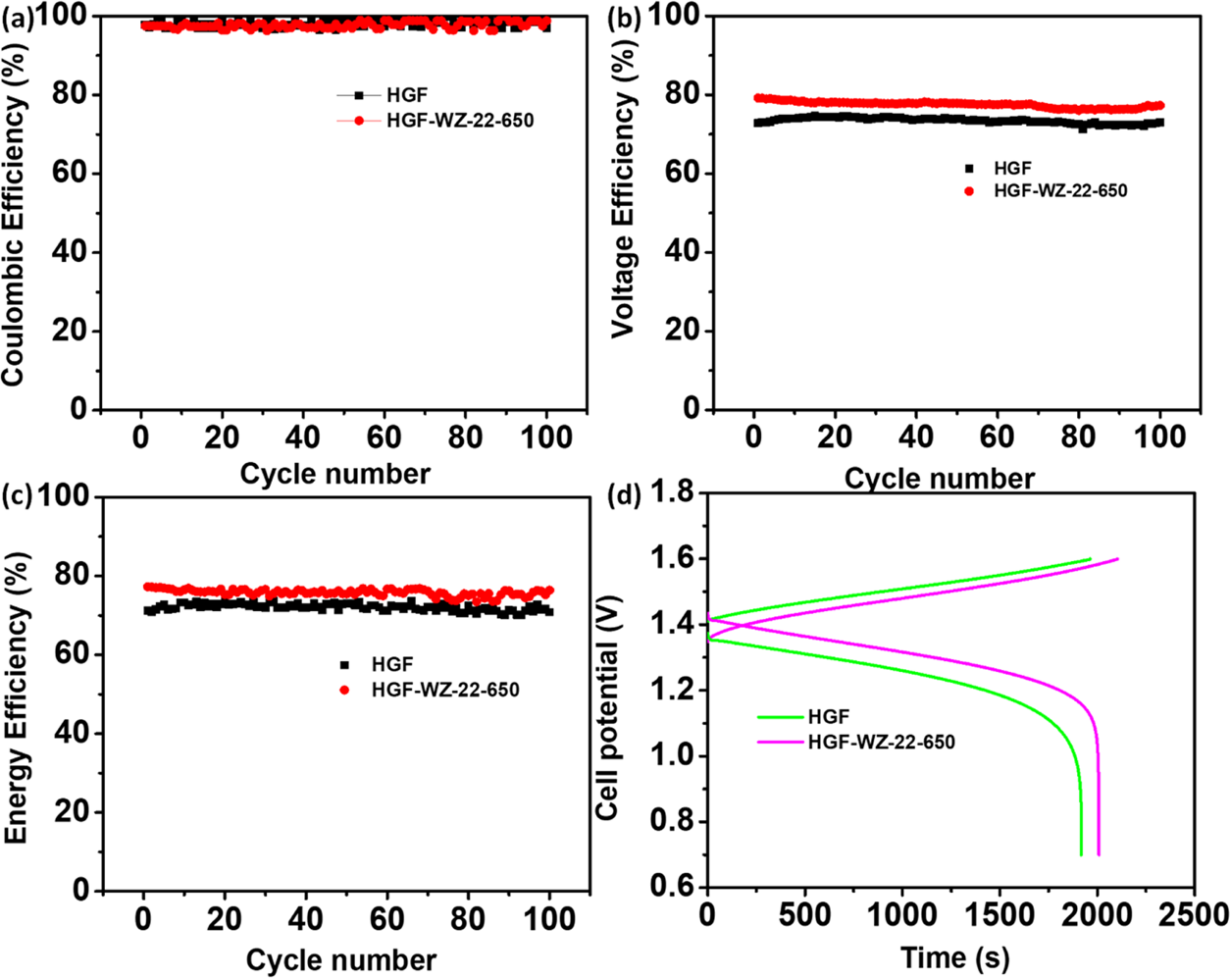
(a) CE, (b) VE, (c) EE, and (d) performance of charge/discharge tests for the cell with HGF and HGF-WZ-22-650.

The stability of the battery with HGF-WZ-22-650 was studied by a 100-lifetime test at 140 mA cm^–2^, as presented in Figure 8. The CE values of the cells built with HGF and HGF-WZ-22-650 electrodes are almost similar, as shown in Figure 8a. This confirms that self-discharge and side reactions similarly affect the HGF and HGF-WZ-22-650 electrodes.^15,44^Figure 8b,c shows the two electrodes’ average VE and EE. The performance for the charge/discharge of the cell tests (Figure 8d). Throughout 100 cycles, the results remain constant for the HGF-WZ-22-650 electrode, ensuring its high chemical stability and electrochemical robustness in an acid vanadium electrolyte. There was no noticeable fading of the efficiencies, indicating that the HGF-WZ-22-650 nanoparticles provided the best stability and electrocatalytic effect by adhering to the GF surface for a long time during repetitive cycling. Moreover, Figure S6 depicts the charging/discharging rate capacity of electrodes prepared at *J* ranging from 80 to 160 mA cm^–2^ and returning to 80 mA cm^–2^. Figure S6a demonstrates nearly identical CE values among individual electrodes at the same current density. However, a high charge/discharge rate can lead to notable increases in both charge and discharge overpotential, alongside substantial reductions in VE and EE, depicted in Figure S6b,c, respectively. Additionally, at all applied currents, the HGF-WZ-22-650 electrode’s discharge capacities are much higher than those of HGF cells, as shown in Figure S6d. Figure S8a–d shows the SEM images of the WZ-22-650 catalyst deposit on the HGF surface, which consists of uniform graphite microfiber. Figure S8a,b shows the morphology of the SEM image that enables clear identification of the WZ-22-650 electrode after and before the CV test, and the EDS of the sample shows all elements anchored in the GF (Figure S4). Further investigation of the morphology before and after the charge/discharge test (Figure S8c,d) and Figure S4 shows the elemental distribution on the surface of the GF. Additionally, the SEM elemental mapping of the HGF-WZ-22-650 electrode is confirmed after numerous charge/discharge cycles, as shown in Figure S5.

The exceptional performance of the WZ-22-650 nanocomposite electrode could be attributed to the following factors: (1) Because MOFs have a regular arrangement of metal nodes and heteroatoms, they are sensitive to the formation of consistently distributed metal species and other dopants.^25^ Many MOF materials demonstrate better catalytic activity from metal ions as a result of catalytic activity arising from molecular moieties. (2) The creation of new W–O–Zr bonds confirms that WO*_x_* is firmly anchored to ZrO_2_, which is critical to facilitating vanadium redox reactions. MOFs possess a stable structure comprising metal-based nodes and a coordination network with organic linkers, including potential vacancies.^4,24^ (3) Inorganic nodes possess catalytic activity by eliminating solvent ligands, leading to coordinately unsaturated metal ion sites that serve as catalytic centers.^26^ These active entities can be integrated into MOFs either during synthesis, by being prelinked on the organic linkers, or after MOF formation through postgrafting onto the framework. Furthermore, the “double solvents” method can be used to immobilize hydrophilic guest species (ammonium meta-tungstate) in the pores of UiO-66 due to its high porosity and hydrophilic surface.^28^

This section provides a summary of different studies, comparing the electrochemical performance of the current material against previously reported metal- or metal-oxide-based materials for VRFBs, as indicated in Table S1. Specifically, the cell utilizing HGF-WZ-22-650 demonstrates outstanding performance with an EE of 83.94% and a VE of 87.76% at 80 mA cm^–2^, surpassing the performance of GF-modified electrodes with other catalysts reported in prior studies.

## 4 Conclusions

A double-solvent impregnation approach was used to synthesize zirconium-based MOFs from UiO-66. The as-obtained WZ has multiple electroactive sites with varying electrochemical performance strengths at moderate concentrations of tungsten and calcination temperatures and has the highest amounts of electroactive species because of highly concentrated amorphous poly-tungsten species. Among all of the samples studied, the WZ shows that electroactive sites demonstrate enhanced catalytic ability toward the redox couple. The WZ catalysts derived from MOFs have higher electrochemical activity for vanadium redox flow batteries because of their high surface areas and electroactive sites. These results demonstrate that WO_*x*_ is firmly anchored to ZrO_2_ and forms new W–O–Zr bonds, which are essential for enhancing the redox reactions of vanadium redox couples. The composite WZ-22-650, particularly tungsten (22%), exhibits the highest electrocatalytic activity toward the redox couple compared with HGF. Charge/discharge tests further confirm that VRFBs using the WZ-22-650 catalyst demonstrate excellent efficiencies. The simple process opens up a new method for making metal-oxide-based catalysts and gives WZ catalysts outstanding catalytic activity and good cyclability.

## Supporting Information Available

Experimental schemes; SEM images; XRD patterns; XPS spectra; EDS; SEM elemental mapping; charge/discharge efficiency and capacity recovery; contact angle measurement; SEM before and after performance; and comparison of electrode performance with previous report (PDF)
