# Supplementary material for: Surface Electroactive Sites of Tungstated Zirconia Catalysts for Vanadium Redox Flow Batteries
Source: ACS Appl Mater Interfaces. 2024 Feb 5;16(6):7047–56. doi: 10.1021/acsami.3c14633 (PMC10875639; doi:10.1021/acsami.3c14633)
Supplement: Supplementary file 1 — am3c14633_si_001.pdf [file am3c14633_si_001.pdf]

Supporting information

Surface Electroactive Sites of Tungstated Zirconia Catalysts for Vanadium Redox Flow Batteries

Aknachew Mebreku Demeku,[1] Daniel Manaye Kabtamu,[#1,2] Guan-Cheng Chen,[1] Yun-Ting Ou,[1] Zih-Jhong Huang,[1] Tai-Chin Chiang,[3] Hsin-Chih Huang,[3] Chen-Hao Wang*,[1,4,5]

[1]*Department of Materials Science and Engineering, National Taiwan University of Science and Technology, Taipei 106335, Taiwan*

[2]*Department of Chemistry, Debre Berhan University, Po.Box: 445, Debre Berhan 00000, Ethiopia*

[3]*Global Development Engineering Program, National Taiwan University of Science and Technology, Taipei 106335, Taiwan*

[4]*Hierarchical Green-Energy Materials (Hi-GEM) Research Center, National Cheng Kung University, Tainan 70101, Taiwan*

[5]*Advanced Manufacturing Research Center, National Taiwan University of Science and Technology, Taipei 106335, Taiwan*

[e]*Advanced Manufacturing Research Center, National Taiwan University of Science and Technology, Taipei 106335, Taiwan*

**Corresponding author:* *chwang@mail.ntust.edu.tw* *(Prof. Chen-Hao Wang)* Fax: +886-2-2737-6544; Tel: +886-2-2730-3715

[#]*Co-corresponding author:* *danielmanaye@gmail.com* *(Dr. Daniel M. Kabtamu)*

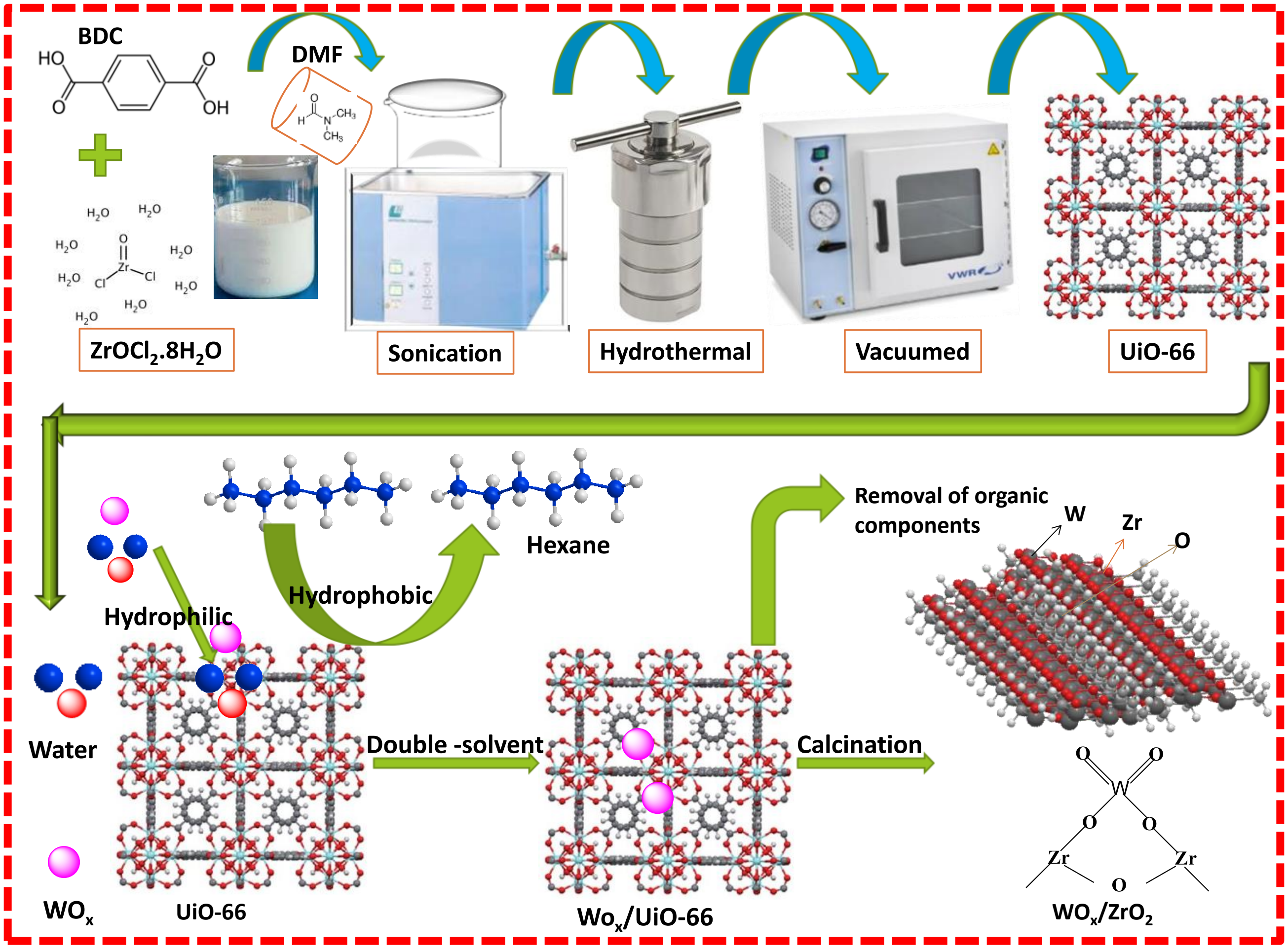

Scheme S1 Synthetic procedure of the double-solvent method and transformation of WO_x/UiO-66 into tungstated zirconia (WZ) by thermal decomposition.

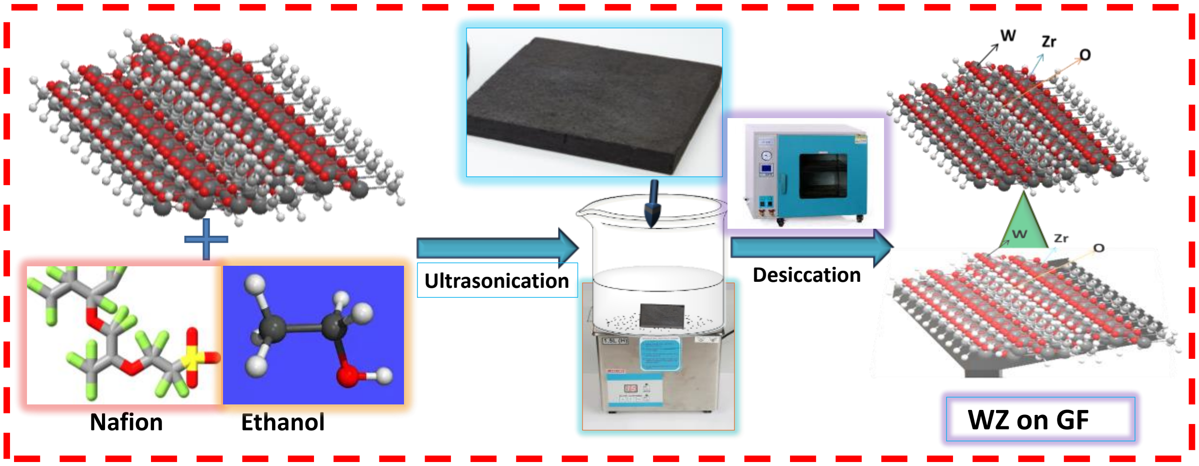

Scheme S2 Schematic diagram for the preparation of high-entropy oxides on graphite felt.

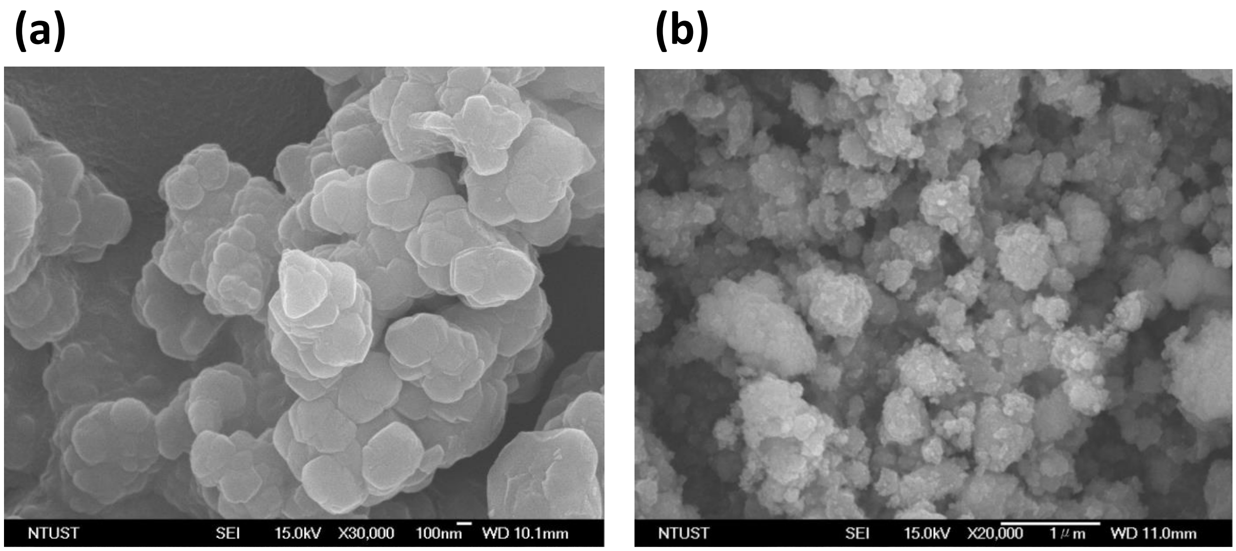

Figure S1 SEM images of the (a) UiO-66 and (b) WZ-22-650.

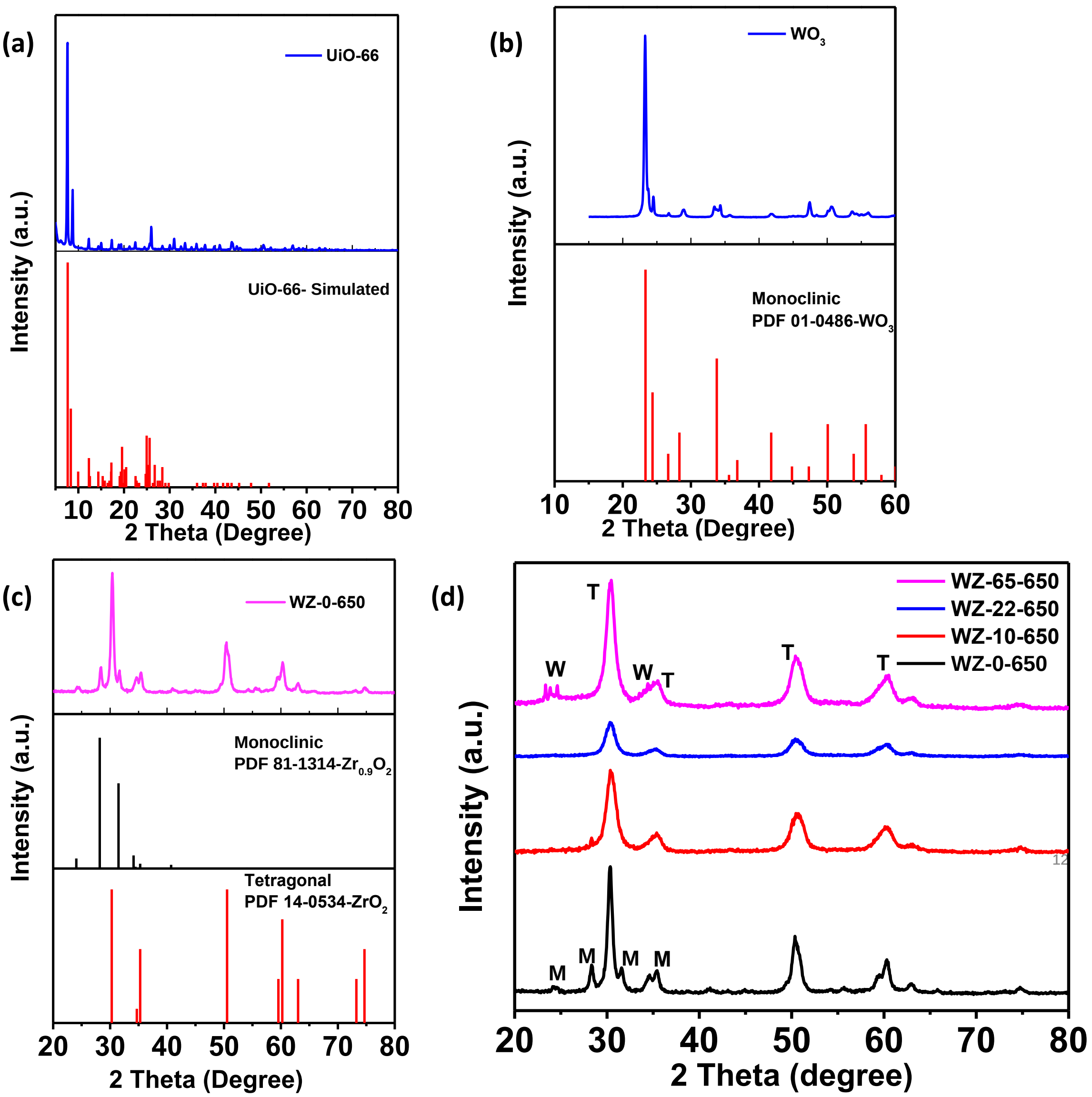

Figure S2 the XRD pattern of (a) UiO-66, (b) WO_3, (c) WZ-0-650, and (d) different W contents.

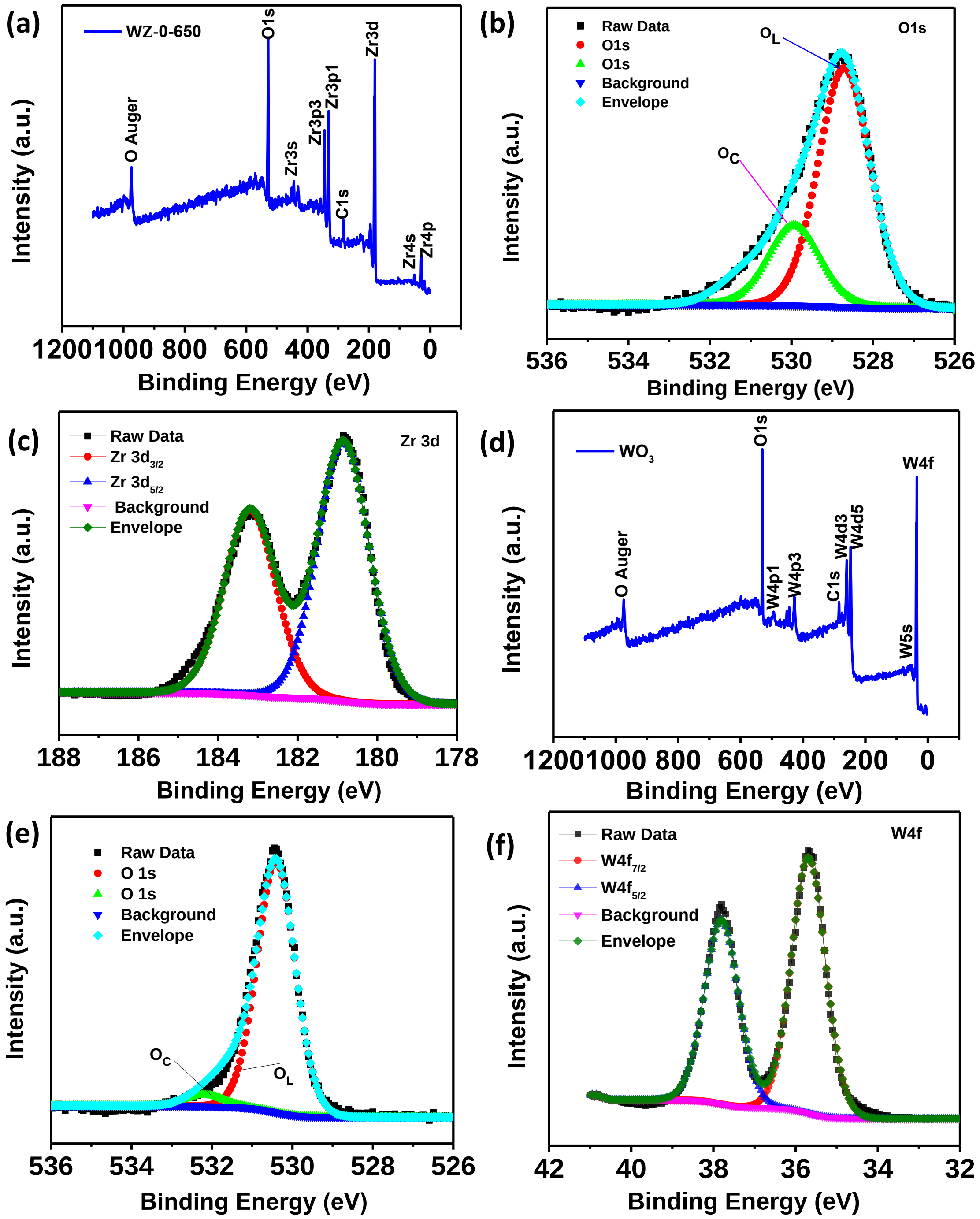

Figure S3 XPS analysis of the WZ-0-650 and WO_3: (a) element survey, (b) O1s, (c) Zr3d, (d) element survey, (e) (O1s), and (f) W4f, respectively.

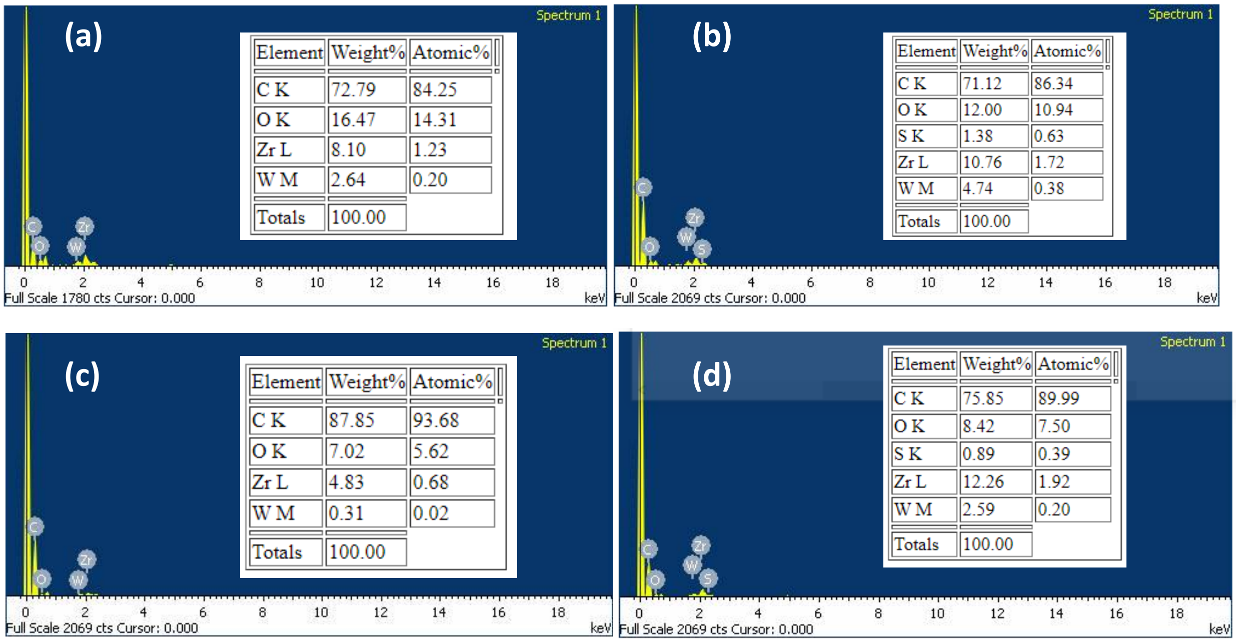

Figure S4 the EDS images of HGF-WZ-22-650 electrode, (a) before CV, (b) after CV, (c) before charge-discharge, and (d) after charge-discharge.

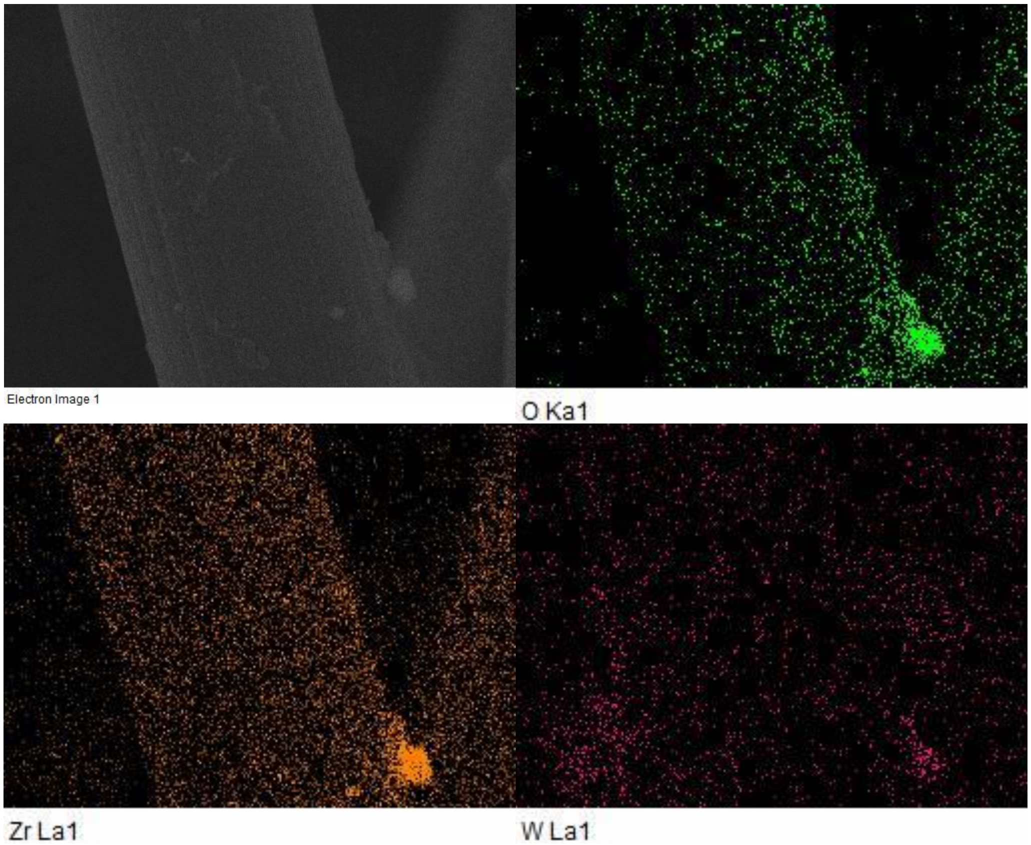

Figure S5 SEM elemental mapping of the HGF-WZ-22-650 electrode after 100 charge-discharge cycles.

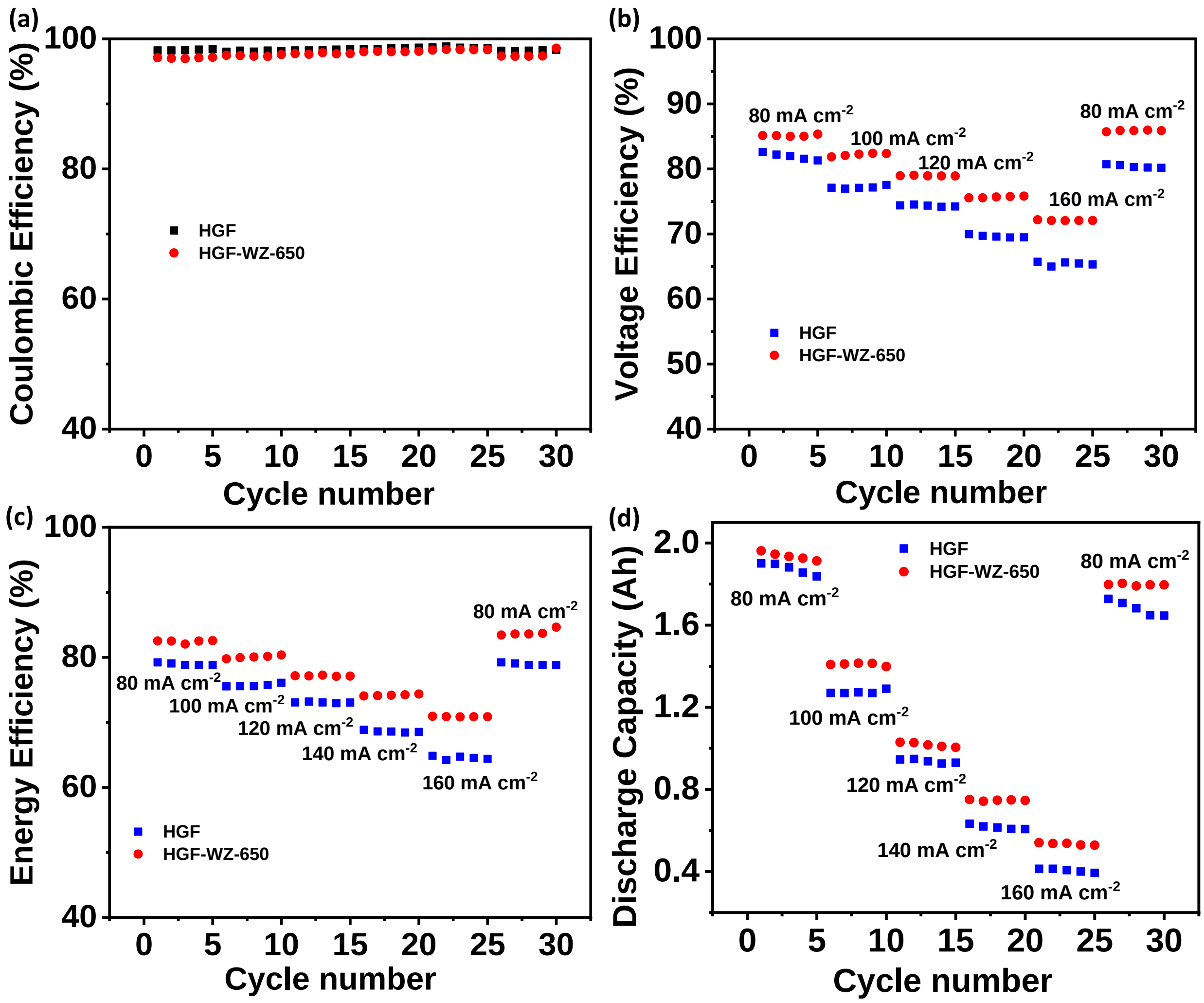

Figure S6 (a) The CE, (b) VE, (c) EE, and (d) discharge capacity of the HGF and HGF-WZ-22-650 electrodes with cycle numbers at different current densities of 80 to 160 mA cm^{-2}.

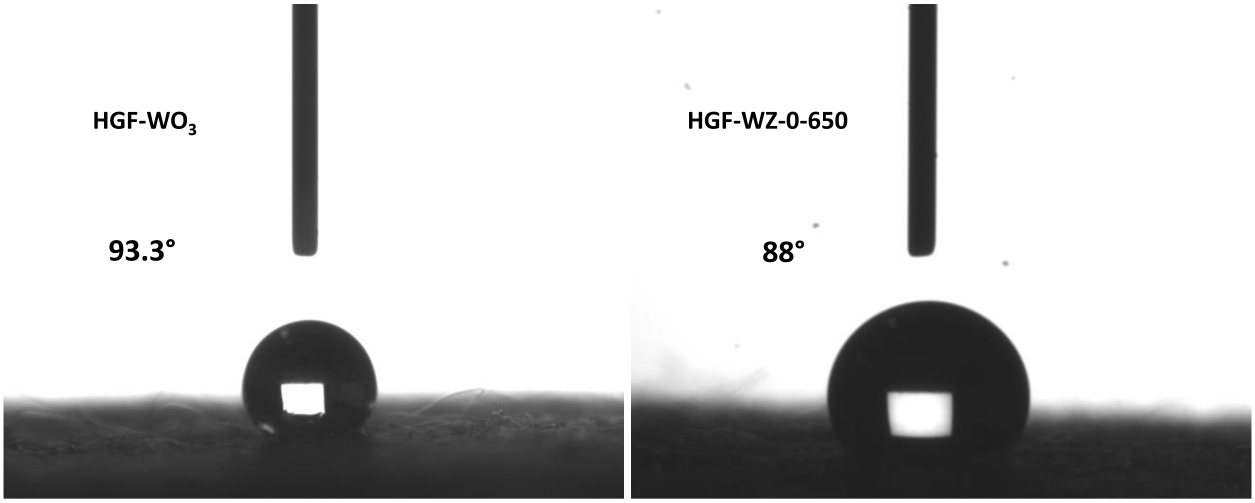

Figure S7 The photographs of the contact angle measurements in (a) $HGF-WO_3$ and (b) HGF-WZ-0-650 electrodes.

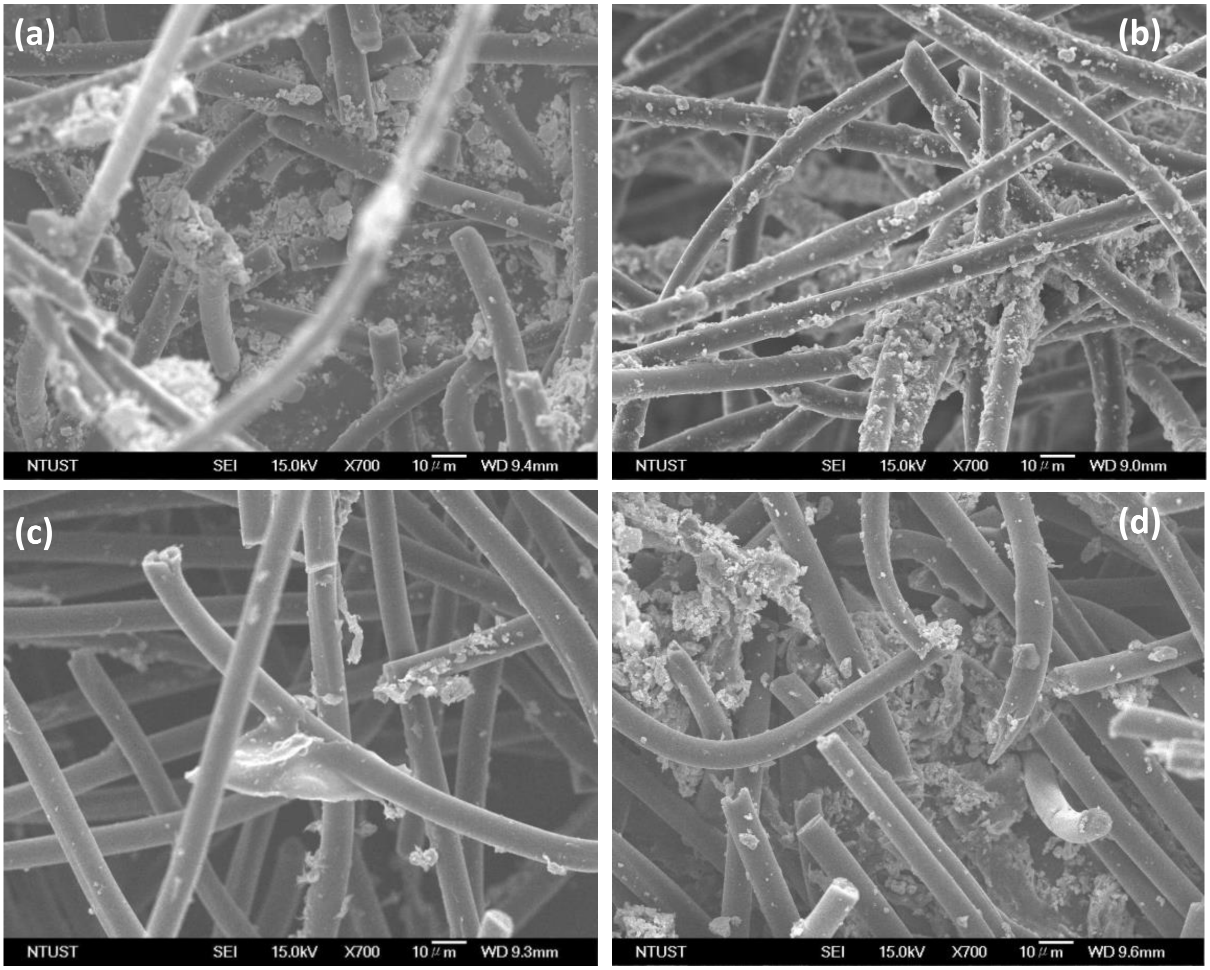

Figure S8 The SEM images (a) before CV, (b) after CV, (c) before charge/discharge, and (d) after charge/discharge of the HGF-WZ-22-650 electrode.

Table S1. Comparison of the CE, VE, and EE of the HGF-WZ-22-650 material with previously reported metal/metal oxide materials for VRFBs Application.

Material	Electrolyte	Current density (mA cm^{-2})	CE (%)	VE (%)	EE (%)	Ref.
HGF-WZ-22-650	1.6 M $VOSO_4$ + 4.6 M H_2SO_4	80	95.65	87.76	83.94	This Work
		160	97.52	76.76	74.86	
$BiVO_4$-GF	1.6 M $VOSO_4$ + 4.6 M H_2 SO_4	100	97.23	77.57	75.42	[1]
SnO_2-P-GF	0.8 M $VOSO_4$ + 3 M H_2 SO_4	150	96.11	69.20	71.90	[2]
Ti/IrO_2 : Ta_2O_5	1.7 M $VOSO_4$ + 4 M H_2SO_4	40	90	91	81.0	[3]
Mn_3O_4/CF	2 M $VOSO_4$ + 2.5 M H_2SO_4	40	83.5	91.0	76.0	[4]
CeO_2/GF	2 M $VOSO_4$ + 2 M H_2SO_4	100	87.9	84.2	74.0	[5]
Nb-doped h-WO_3 NWs/GF	1.6 M $VOSO_4$ + 2.5 M H_2SO_4	80	93.16	83.83	78.10	[6]
WO_3 /GF	1 M $VOSO_4$ + 3 M H_2 SO_4	100	99.7	72.2	72	[7]
PbO_2 /GF	0.5 M $VOSO_4$ + 3 M H_2 SO_4	80	99.7	78.3	78.1	[8]
H−CeO_2NWs−GF	1.6 M $VOSO_4$ + 2.5 M H_2SO_4	80	93.6	81.7	76.84	[9]
TNO-GF	1.6 M $VOSO_4$ + 2.5 M H_2SO_4	100	95.52	82.77	79.06	[10]

References

(1) Kabtamu, D. M.; Li, Y.-Z.; Bayeh, A. W.; Ou, Y.-T.; Huang, Z.-J.; Chiang, T.-C.; Huang, H.-C.; Wang, C.-H. BiVO4-Decorated Graphite Felt as Highly Efficient Negative Electrode for All-Vanadium Redox Flow Batteries. *ACS Applied Energy Materials* **2023**, *6* (6), 3301-3311.

(2) Feng, X.; Zhang, Z.; Zhang, T.; Xue, J.; Han, C.; Dai, L.; Wang, L.; He, Z. Enhanced Catalysis of P-doped SnO2 for the V2+/V3+ Redox Reaction in Vanadium Redox Flow Battery. *Frontiers in Chemistry* **2021**, *9*, 688634.

(3) Raghu, S. C.; Ulaganathan, M.; Lim, T. M.; Kazacos, M. S. Electrochemical behaviour of titanium/iridium (IV) oxide: Tantalum pentoxide and graphite for application in vanadium redox flow battery. *Journal of power sources* **2013**, *238*, 103-108.

(4) Kim, K. J.; Park, M.-S.; Kim, J.-H.; Hwang, U.; Lee, N. J.; Jeong, G.; Kim, Y.-J. Novel catalytic effects of Mn 3 O 4 for all vanadium redox flow batteries. *Chemical Communications* **2012**, *48* (44), 5455-5457.

(5) Zhou, H.; Xi, J.; Li, Z.; Zhang, Z.; Yu, L.; Liu, L.; Qiu, X.; Chen, L. CeO 2 decorated graphite felt as a high-performance electrode for vanadium redox flow batteries. *Rsc Advances* **2014**, *4* (106), 61912-61918.

(6) Kabtamu, D. M.; Chen, J.-Y.; Chang, Y.-C.; Wang, C.-H. Electrocatalytic activity of Nb-doped hexagonal WO 3 nanowire-modified graphite felt as a positive electrode for vanadium redox flow batteries. *Journal of Materials Chemistry A* **2016**, *4* (29), 11472-11480.

(7) Shen, Y.; Xu, H.; Xu, P.; Wu, X.; Dong, Y.; Lu, L. Electrochemical catalytic activity of tungsten trioxide-modified graphite felt toward VO2+/VO2+ redox reaction. *Electrochimica Acta* **2014**, *132*, 37-41.

(8) Wu, X.; Xu, H.; Lu, L.; Zhao, H.; Fu, J.; Shen, Y.; Xu, P.; Dong, Y. PbO2-modified graphite felt as the positive electrode for an all-vanadium redox flow battery. *Journal of Power Sources* **2014**, *250*, 274-278.

(9) Bayeh, A. W.; Lin, G.-Y.; Chang, Y.-C.; Kabtamu, D. M.; Chen, G.-C.; Chen, H.-Y.; Wang, K.-C.; Wang, Y.-M.; Chiang, T.-C.; Huang, H.-C. Oxygen-vacancy-rich cubic CeO2 nanowires as catalysts for vanadium redox flow batteries. *ACS Sustainable Chemistry & Engineering* **2020**, *8* (45), 16757-16765.

(10) Kabtamu, D. M.; Bayeh, A. W.; Chiang, T.-C.; Chang, Y.-C.; Lin, G.-Y.; Wondimu, T. H.; Su, S.-K.; Wang, C.-H. TiNb2O7 nanoparticle-decorated graphite felt as a high-performance electrode for vanadium redox flow batteries. *Applied Surface Science* **2018**, *462*, 73-80.
